# Fabricating PES/SPSF membrane *via* reverse thermally induced phase separation (RTIPS) process to enhance permeability and hydrophilicity

**DOI:** 10.1039/c9ra05707b

**Published:** 2019-08-27

**Authors:** Sheng-Hui Liu, Hang Yang, Shi-Feng Ji, Chun-Mei Gao, Han Fang, Yun-Qing Xing, Nai-Xu Han, Guo-Dong Ding, Lei Jia

**Affiliations:** College of Marine Ecology and Environment, Shanghai Ocean University Shanghai 201306 China sfji@shou.edu.cn cmgao@shou.edu.cn +86-21-61908337 +86-21-61900330; Center for Polar Research, Shanghai Ocean University Shanghai 201306 China; Marine Environment Monitoring and Assessment Center, Shanghai Ocean University Shanghai 201306 China; Shanghai Environmental Protection Co., Ltd. Shanghai 200003 China

## Abstract

A new method was presented to prepare hydrophilic PES/SPSF flat-sheet membrane by a reverse thermally induced phase separation (RTIPS) method to enhance permeability and hydrophilicity. SPSF was self-made and was blended to improve the hydrophilicity of PES flat-sheet membrane. The performance of PES/SPSF flat-sheet membrane, which varied with SPSF content and coagulation water bath temperature, was investigated by SEM, FTIR, AFM, pure water flux, BSA rejection rate, water contact angle and long-term testing. FTIR results proved the successful blending of SPSF with PES membrane, SEM images showed that dense skin surface and finger-like structure emerged in the membrane fabricated by NIPS method, while a porous top surface and sponge-like structure emerged in the membrane fabricated by RTIPS. The pure water flux and BSA rejection rate of the membrane for RTIPS were both higher than those for NIPS. AFM images revealed that surface roughness increased with the addition of SPSF. The water contact angle decreased with the increase of SPSF, which illustrated better hydrophilicity with the addition of SPSF. The flat-sheet PES membrane prepared with 2 wt% SPSF by RTIPS method exhibited decent properties, reaching maximum pure water flux (966 L m^−2^ h^−1^) and at the same time the BSA rejection rate was 79.2%. The long-term test proved that the anti-fouling performance of PES/SPSF membrane was better than that of PES membrane.

## Abstract 2

A new method is presented to prepare hydrophilic PES/SPSF flat-sheet membrane by a reverse thermally induced phase separation (RTIPS) method to enhance permeability and hydrophilicity.

## Graphical abstract

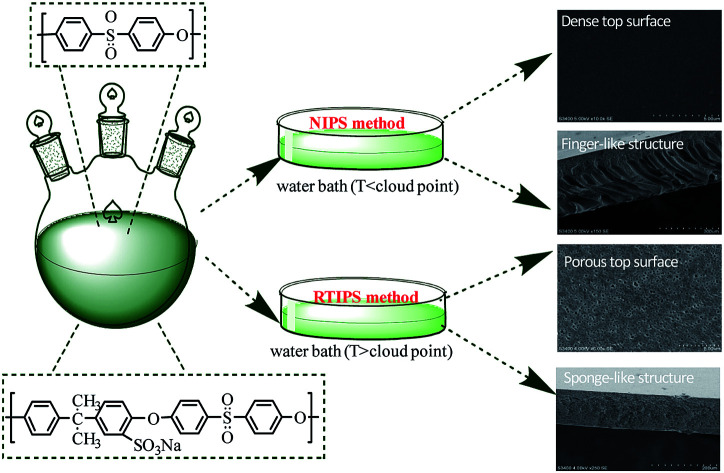

## 1 Introduction

Membrane technology has been widely used in solving deteriorating water quality caused by the appearance of both conventional pollutants as well as new pollutants in water.^1^ However, the membrane properties trend to be limited largely by membrane morphology^2^ and fouling.^3^ Owing to the deposition of organic, biological and colloidal matters on the membrane and within the membrane pores, the membrane morphology (top surface or cross-section) and hydrophilicity play a key role during the separation procedure. Therefore, by modifying membrane morphology^4^ and improving hydrophilicity^5^ can minimize membrane fouling.

The non-solvent induced phase separation (NIPS) method^6,7^ and thermally induced phase separation (TIPS) process,^8,9^ which are based on mass transfer and heat transfer, respectively, are the two main ways to prepare membranes. However, finger-like structures and dense skin surface in the membrane limit the wide application of the NIPS method, and high process temperature and few diluents hinder the extensive use of TIPS method. As a new membrane preparation technology, reverse thermally induced phase separation (RTIPS) process^10^ has received unprecedented attention since it combines both advantages of NIPS and TIPS process. Compared with NIPS method, the advantages of RTIPS method are that it turns finger-like structure into sponge-like structure as well as dense skin top surface transforms into uniform porous surface. Uniform porous surface contribute to high pure water flux, and sponge-like structure would be propitious to high BSA rejection rate and excellent mechanical properties. Compared with TIPS method, the advantages of RTIPS method are that the RTIPS process temperature and energy consumption are lower than TIPS process. Zhao *et al.*^11^ prepared PSF/HBPE hollow fiber membrane by RTIPS process, the effects of preparation methods (NIPS or RTIPS method) and the mass ratio of HBPE on the structure and properties of PSF membranes were studied, and found that finger-like cross-section appeared in the membrane by NIPS method, and sponge-like cross-section emerged in the membrane by RTIPS process, both antifouling property and water permeate rate were improved with the addition of HBPE in a great extent. Generally, RTIPS method generates less pollution to the environment and is a low energy consumption process, these properties make it appropriate for industrialization.

One shortcoming of PES membrane is its hydrophobicity in nature,^12,13^ which can often results in serious membrane fouling and the permeability decline during the practical applications in water treatment. Therefore modification is necessary. Among the modification methods, polymer blending is the most common and simple method to improve the hydrophilicity of the membrane,^14–16^ in which the compatibility between the polymers is an important factor affecting the structure and properties of the blend membranes, poor compatibility between polymers would lead to not only uneven membrane surface, but also the membrane with stripes and macroporous structure as result of poor membrane properties.^17^ Therefore, suitable hydrophilic substances are prerequisite for hydrophilic modification, many hydrophilic substances are blended into the PES membrane to improve the hydrophilicity of PES membrane, such as: hydroxyapatite nanotubes,^18^ oxygen-doped graphitic carbon nitride,^19^ CuO,^20^ cellulose acetate^21^ and so on. However, the nature of these substances (hydrophilic) and the polyethersulfone bulk properties (hydrophobicity) are opposite, although the hydrophilicity of the PES membrane is improved, the stability of the casting solution system is poor, which easily lead to poor PES membrane properties due to the reverse nature between the polymers. The method to overcome this problem is to form the specific interactions between polymers such as blending hydrophilic substance which containing a similar group with polymers. Ma *et al.*^22^ prepared PES/SPSF blend membranes with different sulfonation degrees by phase inversion process, the effect of polymer concentration and additives of casing solution on the performance of PES/SPSF blend membranes were investigated, the results demonstrated that the mutual compatibility between SPSF and PES was good, however, a low water permeate flux (27.2 L m^−2^ h^−1^) was ascribed to the dense skin surface and finger-like asymmetrical structure of the blend membrane by phase inversion process. Moreover, dense skin surface and finger-like asymmetrical structure of the blend membranes can be enhanced from the reverse thermally induced phase separation process. Therefore, the effect of the addition of SPSF and membrane formation mechanism on membrane properties and morphology still need to be further studied.

The objective of this study is to investigate the effect of SPSF on the compatibility, morphologies and properties of PES/SPSF membranes as well as the influence of RTIPS process on the morphologies and properties of PES/SPSF membranes. Hereon, PES/SPSF blend membranes were prepared from PES/SPSF/DMAc/DEG casting solution with water as coagulation bath *via* NIPS or RTIPS method. The morphologies and properties of the PES/SPSF membranes were characterized by scanning electron microscopy (SEM), pure water flux, bovine serum albumin (BSA) rejection, atomic force microscope (AFM) and water contact angle.

## 2 Experimental

### 2.1 Materials

Polyethersulfone (PES, *M*_w_ = 45 000) and polysulfone (PSF) were acquired from BASF Co. Ltd. (Germany). *N*,*N*-Dimethyl acetamide (DMAc), diethylene glycol (DEG), chloroform, trimethylsilyl chlorosulfonate (Aldrich) and sodium methoxide were purchased by Shanghai Chemical Reagent Co. Ltd. Bovine serum albumin (BSA, *M*_w_ = 67 000) was supplied by Shanghai Lianguan Biochemical Engineering Co. Ltd. Pure water was self-made.

### 2.2 Preparation of SPSF

5 g polysulfone dissolved in 100 mL chloroform at room temperature and handled with trimethylsilyl chlorosulfonate to generate a silyl sulfonate polysulfone intermediate. The molar ratio of sulfonating agent to polymer-repeat units determined the amount of intermediate produced. A slightly excessive sodium methoxide was then added into the solution to decompose the silyl sulfonate intermediates and generate final sulfonated product. All samples were cleaned thoroughly with methanol, rinsed some times with deionized water and dried in vacuum oven at 100 °C for 1 d.

The sulfonation degree (DS) was expressed by ion exchange capacity (IEC) of the prepared SPSF. The IEC was calculated by adding 0.3 g SPSF in 30 mL 1 mol L^−1^ NaCl liquor about 24 h to release the hydrogen ions and titration was carried out in standardized 0.1 mol L^−1^ NaOH solution with phenolphthalein as an indicator.1
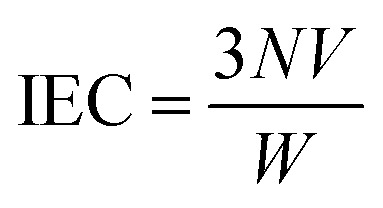

2 DS = (IEC × 442/1000) × 100%

where *N* is the equivalent concentration of sodium hydroxide standard solution, *V* is the volume of consumed sodium hydroxide standard solution, *W* is the mass of sulfonated polysulfone.

### 2.3 Preparation of cast solution

**Fig. 1:**
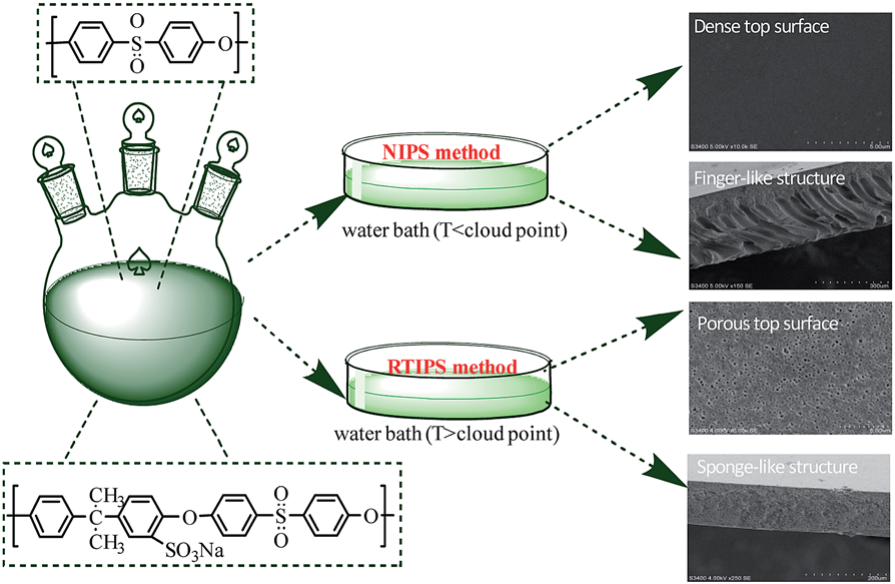
Schematic illustration for the preparation of PES/SPSF membrane by NIPS and RTIPS method.

*N*,*N*-Dimethyl acetamide (DMAc) and diethylene glycol monocondensate (DEG) were premixed primarily as mixed solvents in a Erlenmeyer flask, then SPSF with different mass percents (0 wt%, 1 wt%, 2 wt%, 3 wt% and 4 wt%) was dissolved in mixed solvents and stirred until SPSF dissolved completely, and measured amounts of PES was dissolved in cast solution and stirred about few hours at room temperature. The prepared cast solutions degassed about 1 d at room temperature for casting. The brief membrane preparation process by NIPS method and RTIPS method was shown in Fig. 1. The composition of cast solution and the temperatures of coagulation water bath were exhibited in Tables 1 and 2, respectively.

**Table 1:** The compositions of cast solution

Cast solution no.	Cast solution composition (wt%)			
	PES	SPSF	DMAc	DEG
MSPSF-0	17.0	0.0	46.1	36.9
MSPSF-1	16.0	1.0	46.1	36.9
MSPSF-2	15.0	2.0	46.1	36.9
MSPSF-3	14.0	3.0	46.1	36.9
MSPSF-4	13.0	4.0	46.1	36.9

**Table 2:** The temperature of coagulation water bath

Membrane no.	Water bath temperature (°C)	Membrane no.	Water bath temperature (°C)
MSPSF-0-25	25	MSPSF-2-60	60
MSPSF-0-60	60	MSPSF-3-60	60
MSPSF-1-60	60	MSPSF-4-25	25
MSPSF-2-25	25	MSPSF-4-60	60

### 2.4 Light transmittance and viscosity measurement

The light transmittance measurement was carried out by a self-made device.^23^ A vertical laser was directed on a glass plate with uniform casting solution, which was immersed in hydrogel bath with different temperature fastly. The light intensity information was caught by a photodetector and was recorded in computer. The precipitation rate of cast solution was characterized by light transmittance curve to immersion time.

The viscosity of the cast solutions with different mass ratio of SPSF were determined by a DV-II+PRO Digital Viscometer (Brookfield, USA) at 25 °C.

### 2.5 Cloud point test

The cloud point measurement was used to determine the phase separation temperature (*i.e.*, LCST, which was entitled as cloud point) of the cast solution.^10^ The cloud point was ascertained by a self-made device, the specific process of cloud point test is as follows: the homogeneous cast solution was placed between two cover glasses, and the cover glasses were heated on a hot stable (KEL-XMT-3100, Shanghai Weitu Optics and Electron Technology Co. Ltd.) from 25 °C to 100 °C at 1 °C min^−1^. A vertical laser was directed on the cover glasses and the light strength was captured by a photodetector, then the light strength was recorded by a computer. The temperature corresponding to the vertical variation of light intensity was considered as cloud point.

### 2.6 Flat-sheet membrane preparation

The homogeneous cast solution was cast on a glass board at ambient temperature, and the glass board with homogeneous cast solution was rapidly immersed in water coagulation bath with different temperature (Table 2). The prepared membrane was placed in fresh deionized water about 3 d and the deionized water exchanged three times 1 d to remove residual solvent completely. Then the prepared membrane dried in air at room temperature for testing.

### 2.7 SEM test

The morphology of flat-sheet membrane was observed by scanning electron microscope (SEM; Nova NanoSEM, USA). The cross-section of flat-sheet membrane was brittled in liquid nitrogen circumstances. All samples were gold-plated under vacuum circumstances about 60 s.

### 2.8 Permeation performance

The permeation properties of flat-sheet membrane was tested by a self-made device.^23^ All tests were put into effect at room temperature with a continuous pressure of 0.1 MPa. The rejection rate was expressed by BSA aqueous solution (300 mg L^−1^). The *J*_w_ and *R*_BSA_ of the flat-sheet membrane were defined by formulas (3) and (4), respectively.3
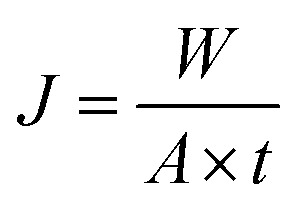
4
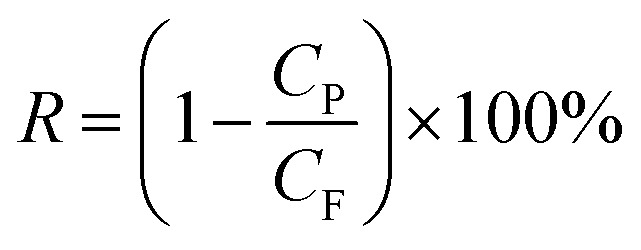
where *J*_w_ is the pure water flux (L m^−2^ h^−1^) of the flat-sheet membrane, *A* is the effective area of the flat-sheet membrane (m^2^), *W* is the bulk of pure water flux (L), *t* is the permeation time (h), *R*_BSA_ is the BSA rejection rate (%), *C*_P_ and *C*_F_ were the concentration of the permeate and feed liquor, respectively.

### 2.9 Antifouling test

To investigate the antifouling performance of the flat-sheet membrane, a cross-flow filtration measurement was made to further detect the long-term antifouling property. The initial pure water flux (*J*_0_) was obtained at 0.1 MPa, after testing 60 min, the pure water measurement was replaced by 0.3 g L^−1^ BSA solution for 60 min running, the contaminated membrane was backwashing with pH = 10 alkali solution after each fouling stage. The filtration process contained three pure water filtration periods and two fouling periods and the pure water flux (*J*_w*i*_, *i* = 1, 2 and 3) was obtained. The flux recovery ratio FRR (%) of the flat-sheet membrane was calculated and defined as following formula (5). The BSA flux of the membranes and the variety of BSA flux with time were also tested.5
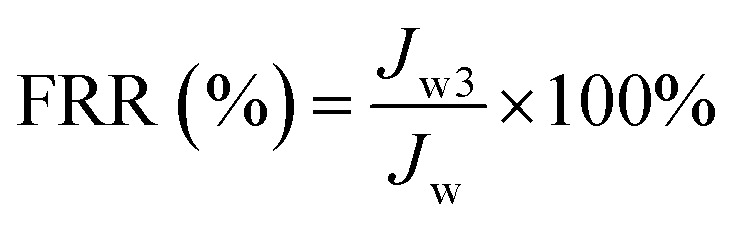


### 2.10 Measurement of porosity and pore size

The porosity *ε* (%) was carried out by the formula as following:6
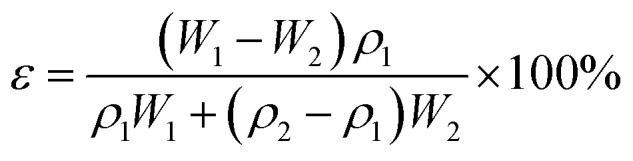
where *ε* is the porosity of the flat-sheet membrane, *W*_1_ and *W*_2_ are the weight of wet membrane and dry membrane, respectively, *ρ*_1_ and *ρ*_2_ are the density of water and PES, respectively.

Mean pore radius *r*_m_ (μm) of the flat-sheet membrane was defined by filtration velocity method referring to the formula of Guerout–Elford–Ferry:^24^7
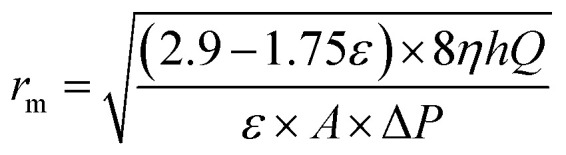
where *η* is the viscosity of water (8.9 × 10^−4^ Pa s^−1^), *h* is the thickness of the membrane (mm), Δ*P* is the operate pressure (0.1 MPa).

Maximum pore size *r*_max_ (μm) of the membrane could be obtained through bubble point procedure referring to the Laplace's equation:^25^8
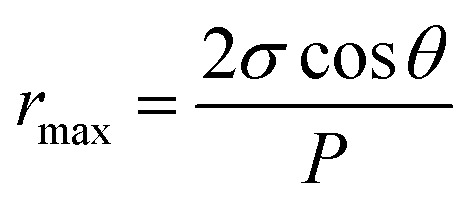
where *σ* is the surface tension of water (22.8 × 10^−3^ N m^−1^), *θ* and *P* are the flat-sheet membrane contact angle (°) and minimum bubble point pressure (MPa), respectively.

### 2.11 Water contact angle test

The hydrophilicity of flat-sheet membrane was tested by water contact angle (*θ*). The water contact angle was measured by the device (JC2000A, Shanghai Zhongcheng Digital Equipment Co. Ltd., China) at room temperature. The image was captured when water droplet contact the membrane surface, then water contact angle samples were taken notes in computer. Each sample was measured three times and then averaged.

### 2.12 AFM test

Atomic force microscopy (Veeco, Nanoscope IIIa Multimode AFM) was to detect the roughness of the flat-sheet membrane. The searching area of the flat-sheet membrane was 20 μm × 20 μm. The roughness of the flat-sheet membrane was analyzed by NanoScope analysis software, the 3D AFM image was drawn by Gwyddion software.

### 2.13 FTIR test

The chemical structure of SPSF and the PES/SPSF flat-sheet membrane were characterized by FTIR (Nicolet 6700, Thermo Electron Scientific Instruments Corp.).

## 3 Results and discussion

### 3.1 The synthetic pathway, sulfonation degree (DS) and FTIR of SPSF

**Fig. 2:**
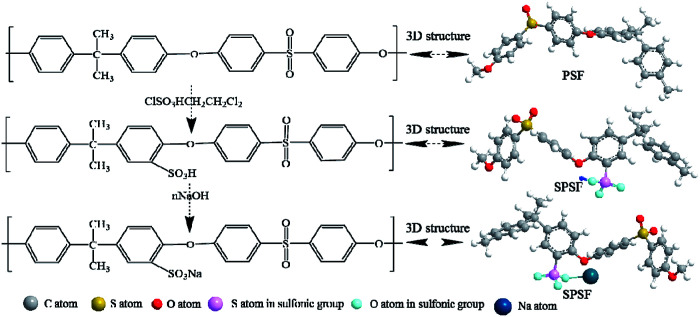
The synthetic route of SPSF.

**Fig. 3:**
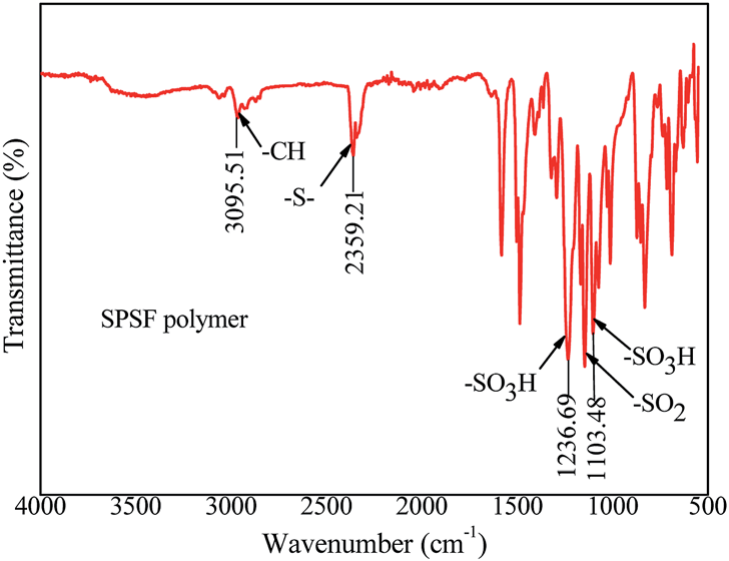
The FTIR spectra of SPSF.

The synthetic pathway and FTIR spectrum of SPSF were displayed in Fig. 2 and 3, respectively. The sulfonation degree of the prepared SPSF was calculated to be 10%. As shown in Fig. 3, the absorption peak at 1103–1236 cm^−1^ represented the symmetrical stretching vibrations of S=O in sulfuric group. In addition, the absorption peak at 2359 cm^−1^ represented the stretching vibrations of –S– in sulfuric group. These results indicated that sulfuric group was successful synthesized in SPSF.

### 3.2 The stability and cloud point of the cast solution

**Fig. 4:**
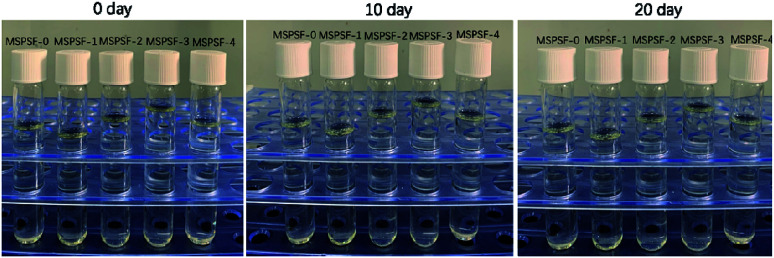
Variation of the cast solution with increase of SPSF content and static time.

**Fig. 5:**
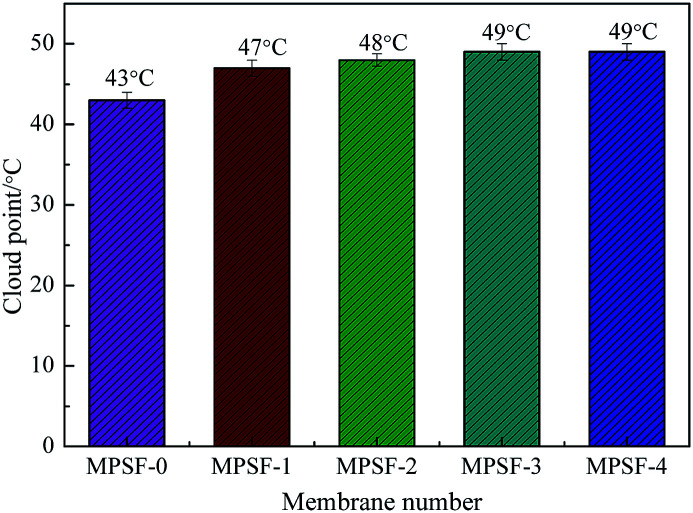
The cloud point of the cast solution with the addition of SPSF.

Cast solutions, as shown in Fig. 4, were transparent with the increase of SPSF content and static time, this phenomenon indicated that good compatibility between SPSF and PES. As exhibited in Fig. 5, the cloud point obtained by heating cast solution from 25 °C to 70 °C at 1 °C min^−1^, the cloud point of MSPSF-0, MSPSF-1, MSPSF-2, MSPSF-3 and MSPSF-4 were 43 °C, 47 °C, 48 °C, 49 °C and 49 °C, respectively. This phenomenon could be explicated by increasing hydrogen bonding between PES/SPSF and mixed solvent when the hydrophilic sulfonic acid group increased in the casting solution. The interaction between PES/SPSF and the mixed solvent became stronger as SPSF content increases, then leaded to phase separation temperature (cloud point) of the LCST cast solution system increased.

However, the growth rate of cloud point slowed down as the concentration of SPSF continued to increase. Due to the good compatibility between PES/SPSF and DMAc, PES and SPSF could dissolve in the mixed solvent (DMAc/DEG) and maintained stability at room temperature for a long time (Fig. 4). When the SPSF content increased, the hydrogen bonding between mixed solvent (DMAc/DEG) and hydrophilic sulfonic acid group was saturated, as SPSF content continued to increase, no more hydrogen bonding formed, although the hydrophilic sulfonic acid group increase, the cloud point was almost constant.

### 3.3 Viscosity and light transmittance

**Fig. 6:**
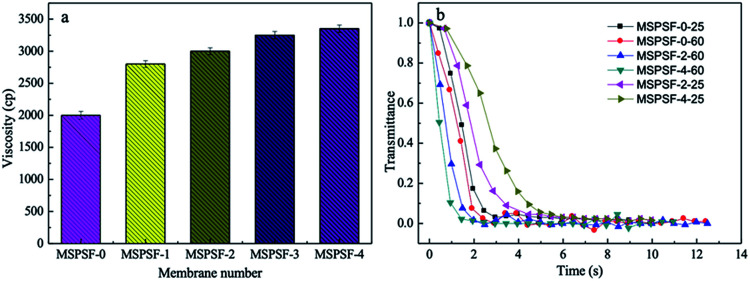
The viscosity (a) and light transmittance curves (b) of the cast solutions.

The viscosity and light transmittance curve of the cast solution were presented in Fig. 6. As shown in Fig. 6a, it could be concluded that the viscosity increased with SPSF content, this phenomenon indicated that SPSF molecules entangled itself with PES molecules in casting solution, which leaded to an increase of initial viscosity.

To illustrate the difference of membrane formation process between NIPS and RTIPS method, light transmittance measurement was carried out. The light transmittance curves descended rapidly at beginning and then turned slowly until unchanged in the end. In Fig. 6b, as for the MSPSF-0-25, MSPSF-2-25 and MSPSF-4-25, the water coagulation bath temperature was lower than the cloud point, the membrane phase separation process followed NIPS method. The descending rate decreased with the increase of SPSF, this could be illustrated by the viscosity of casting solution increased with the addition of SPSF.

As for the MSPSF-0-60, MSPSF-2-60 and MSPSF-4-60, the water coagulation bath temperature was higher than cloud point, the dominant process was RTIPS process. The descending rate increased with the addition of SPSF. This phenomenon indicated that RTIPS process was the dominating process and verificated that heat transfer rate was much faster than mass transfer speed,^26^ the temperature difference between water coagulation bath temperature and cloud point decreased with the addition of SPSF when the water coagulation bath temperature fixed at 60 °C, lower temperature difference contributed to lower heat transfer rate. Generally, high viscosity^27^ and lower temperature difference would decrease precipitation speed, however, phase separation rate increased with the increased SPSF content, this could be explained by more hydrophilic groups (–SO_3_H) with the increased SPSF content, the effect of hydrophilic groups were greater than viscosity and temperature difference, then speeded the phase separation process.

### 3.4 ATR-FTIR analysis of PES/SPSF membrane

**Fig. 7:**
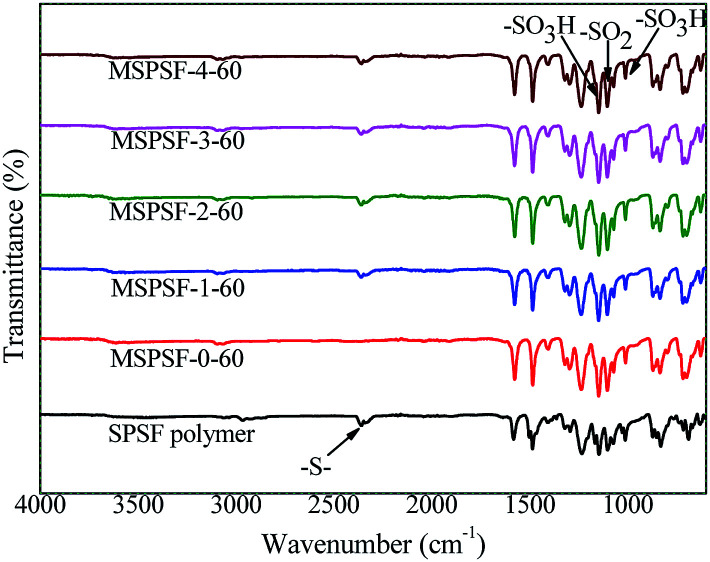
The FTIR spectra of the PES/SPSF membrane.

In order to study the functional groups appeared in PES/SPSF membrane surface and if SPSF was successfully blended into PES membrane, ATR-FTIR analysis was carried out. As shown in Fig. 7, all spectra showed distinctive peaks at 3095 cm^−1^ corresponding to –C–H stretching vibration, 2359 cm^−1^ for –S– stretch, 1236 cm^−1^ and 1103 cm^−1^ for the –SO_3_H of the SPSF and 1147 cm^−1^ corresponding to the stretching of –SO_2_. Moreover, in the same range 1235–1250 cm^−1^, 1000–1072 cm^−1^ and 1119–1150 cm^−1^ corresponding to the asymmetric and symmetric stretching of –SO_3_H and O=S=O groups of SPSF polymer, all the FTIR spectra of PES/SPSF membranes exhibited –S– functional group peak in the same region (2359 cm^−1^), which was absent in the spectrum of pristine PES membrane. This confirmed successful incorporation of SPSF and interaction between SPSF and PES flat-sheet membrane.

### 3.5 Membrane morphology

**Fig. 8:**
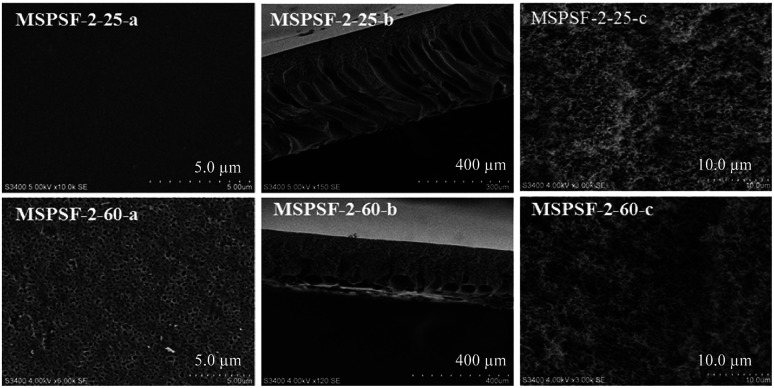
SEM micrographs of MSPSF-2 by NIPS and RTIPS method.

**Fig. 9:**
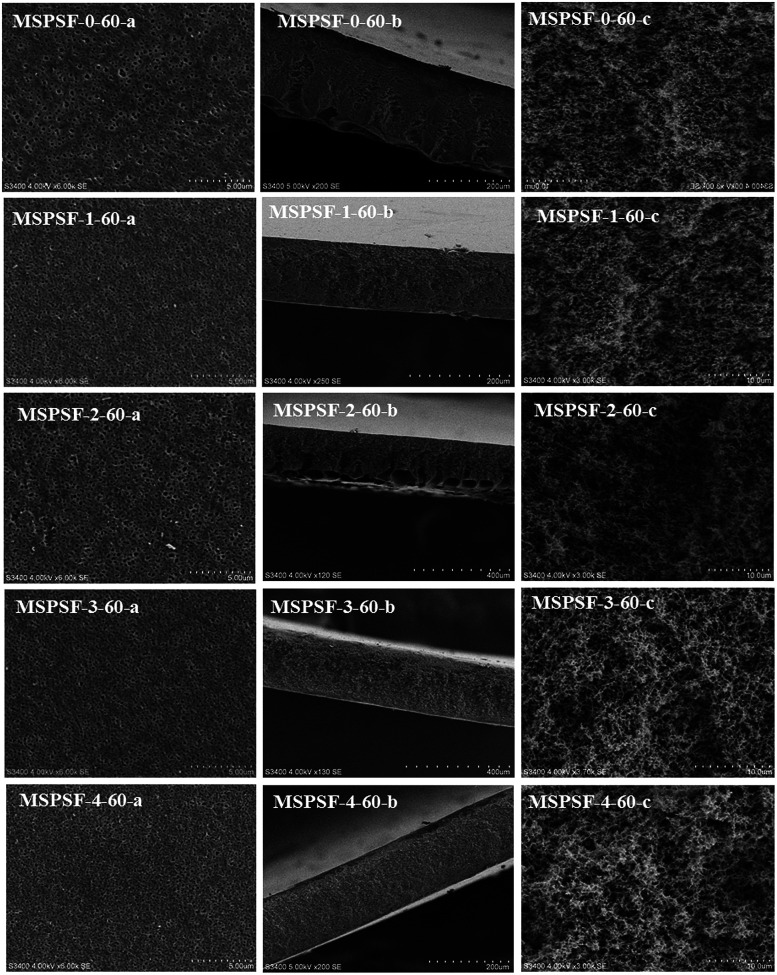
SEM micrographs of the membranes with different SPSF contents. (a) Enlarged top surface; (b) full cross-section; (c) enlarged cross-section.

SEM images of the PES/SPSF flat-sheet membranes were shown in Fig. 8 and 9. The cloud point of MSPSF-0 was 43 °C. When the coagulation water bath temperature was 25 °C (lower than the cloud point), the major driving force of membrane formation mechanism was NIPS process. As for MSPSF-0-25, dense skin layer and finger-like structure were formed. When the coagulation water bath temperature was 60 °C (higher than the cloud point), the membrane formation process was dominated by RTIPS process. As shown in Fig. 8, a bi-continuous cross-section and homogeneous porous top surface in MSPSF-2-60 were obtained, which was the sign of high pure water flux and good mechanical property of the membrane. These observations indicated that instantaneous phase separation happened in NIPS process as well as dense skin surface and finger-like structure occurred in the membrane, while homogeneous porous top surface and sponge-like structure appeared in the membrane by RTIPS method.

Fig. 9 showed the SEM morphology of flat-sheet membrane with different SPSF content when coagulation water bath temperature was fastened at 60 °C. As shown in Fig. 5, the cloud point of the cast solution with different SPSF content was between 47 °C and 49 °C, so the membrane formation mechanism was dominated by RTIPS method when the membrane phase separation temperature was 60 °C. The main driving force of NIPS method and RTIPS method were mass transfer and heat transfer, respectively. Due to the main driving force of the phase separation was heat transfer instead of mass transfer, as shown in Fig. 9, the morphology of MSPSF-0-60, MSPSF-1-60, MSPSF-2-60, MSPSF-3-60 and MSPSF-4-60 were all bi-continuous cross-section and homogeneous porous top surface structure. SEM image of MSPSF-0-60-b, as presented in Fig. 9, exhibited a dense sponge-like cross-section, this phenomenon indicated a rapid exchange speed during the membrane formation procedure because of the lower viscosity. To investigate the effect of SPSF on the flat-sheet membrane morphology, SEM images, as shown in Fig. 9, turned dense sponge-like cross-section into sponge-like structure with the increased SPSF content. This finding was due to the crosslinking structure between SPSF and PES chains, more heat was needed to wreck the interaction during the phase separation, which retarded the phase separation speed and then sponge-like structure emerged in the membrane. Finger-like cross section structure appeared in the bottom of MSPSF-2-60-b, this was attributed to the increased hydrophilic sulfonic group with the addition of SPSF, which accelerated the phase separation process, then leaded to the formation of finger-like structure. The number of pores in PES/SPSF membrane top surface were more than that in PES membrane, while the size of pores were smaller than that in PES membrane. As for MSPSF-1-60, MSPSF-2-60, MSPSF-3-60 and MSPSF-4-60, the holes on the top surface first became bigger then became smaller with the increased SPSF content. These phenomena were consistent with the pure water flux and BSA rejection rate in Fig. 12.

**Fig. 10:**
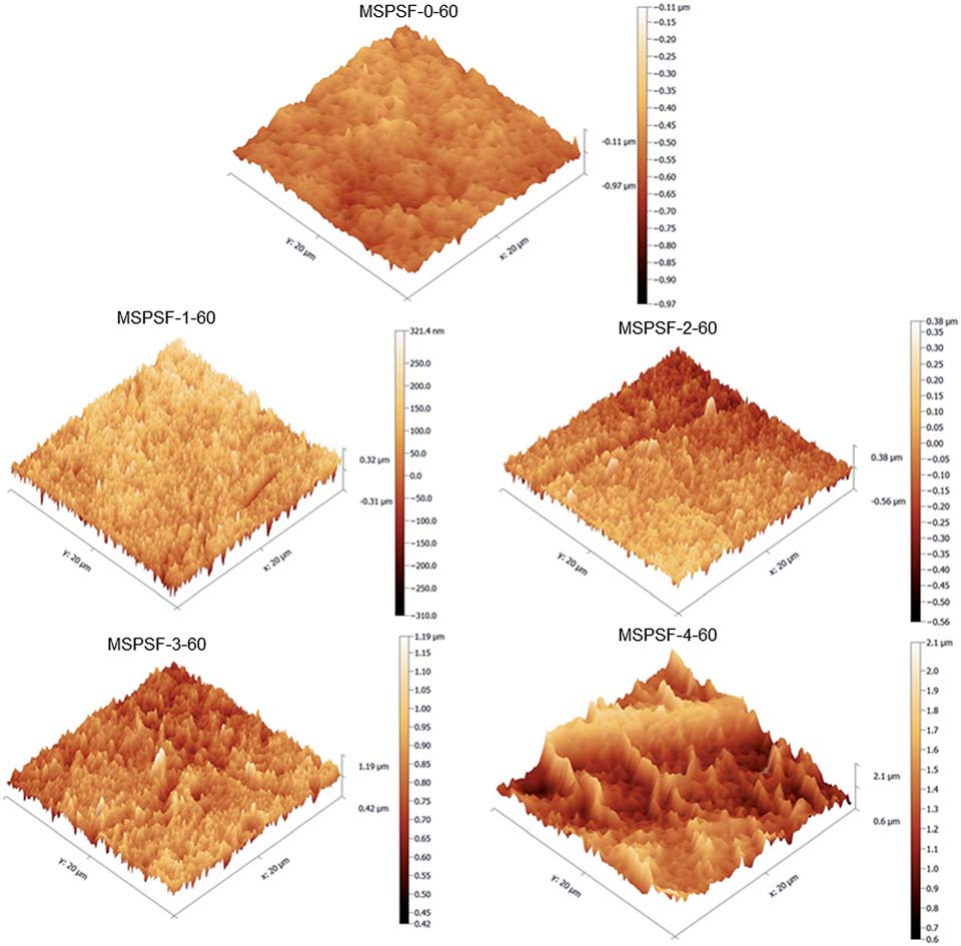
The AFM images of the membrane with different SPSF content.

The membrane surface topography of PES/SPSF flat-sheet membrane with different SPSF content was illustrated in Fig. 10. In the scan area range 20 μm × 20 μm, the *R*_a_ of MSPSF-0-60, MSPSF-1-60, MSPSF-2-60, MSPSF-3-60 and MSPSF-4-60 were 46.2 nm, 68.3 nm, 76.1 nm, 83.7 nm and 104.6 nm, respectively. It could be seen that the membrane surface roughness, especially the surface roughness value of the pristine PES membrane (MSPSF-0-60) was lower than the PES/SPSF membranes. This could be explained by the presence of hydrophilic SPSF which migrated spontaneously to the interface of the flat-sheet membrane and then generated rougher membrane top surface. It could also be observed that the surface roughness values of the PES/SPSF flat-sheet membrane increased with the increased SPSF content. An increase in the flat-sheet membrane roughness indicated an increase in effective membrane surface area causing by the ridges and valleys of nodular shapes, which is beneficial in the improvement of flux.^28^

### 3.6 Pore size and porosity

The porosity and pore size of flat-sheet membrane were listed in Table 3, in which the content of SPSF and coagulation water bath temperature were varied. For membranes prepared by NIPS method (MSPSF-0-25, MSPSF-2-25, MSPSF-4-25), the porosity increased with the content of SPSF. The reason was that the pore gap and hydrophilicity increased with the addition of SPSF. With regard to the membranes by RTIPS method, the porosity first increased and then changed a little with the increase of SPSF content in the following order: MSPSF-0-60, MSPSF-1-60, MSPSF-2-60, MSPSF-3-60, MSPSF-4-60. This phenomenon indicated that the porosity of the membrane by RTIPS was higher than NIPS method, the porosity reached the maximum and then the growth rate slowed down with the increased SPSF. As for MSPSF-2-25 (by NIPS method) and MSPSF-2-60 (by RTIPS method), the porosity of the MSPSF-2-60 was higher than MSPSF-2-25, this phenomenon was consist with the dense skin surface by NIPS method and homogeneous porous surface by RTIPS method.

**Table 3:** Pore size and porosity of PES/SPSF flat-sheet membrane

Membrane no.	SPSF (wt%)	Coagulation bath temperature (°C)	Porosity (%)	*r*_max_ (μm)	*r*_m_ (μm)
MSPSF-0-25	0	25	78.2 ± 0.3	0.237 ± 0.031	0.036 ± 0.003
MSPSF-0-60	0	60	80.9 ± 0.5	0.394 ± 0.005	0.054 ± 0.001
MSPSF-1-60	1.0	60	85.1 ± 0.1	0.302 ± 0.003	0.091 ± 0.004
MSPSF-2-25	2.0	25	83.4 ± 0.5	0.457 ± 0.011	0.052 ± 0.005
MSPSF-2-60	2.0	60	86.4 ± 0.2	0.723 ± 0.031	0.102 ± 0.003
MSPSF-3-60	3.0	60	86.7 ± 0.2	0.717 ± 0.023	0.098 ± 0.004
MSPSF-4-25	4.0	25	85.3 ± 0.2	0.588 ± 0.021	0.061 ± 0.002
MSPSF-4-60	4.0	60	87.2 ± 0.2	0.580 ± 0.009	0.097 ± 0.007

The maximum pore size (*r*_max_) and mean pore size (*r*_m_) of flat-sheet membrane prepared by RTIPS method (MSPSF-2-60) were both higher than that by NIPS method (MSPSF-2-25), this phenomenon consistent with the morphology obtained by NIPS and RTIPS method (Fig. 8). For the membranes prepared by RTIPS method, the *r*_m_ and *r*_max_ reached the maximum at MSPSF-2-60, this appearance was consistent with the variation of pure water flux in Fig. 12. When the mass ratio of SPSF was higher than 2 wt%, the *r*_m_ and *r*_max_, especially the *r*_max_, showed a decreasing trend, this was attributed to the increased viscosity with the addition of SPSF.

### 3.7 Hydrophilicity

**Fig. 11:**
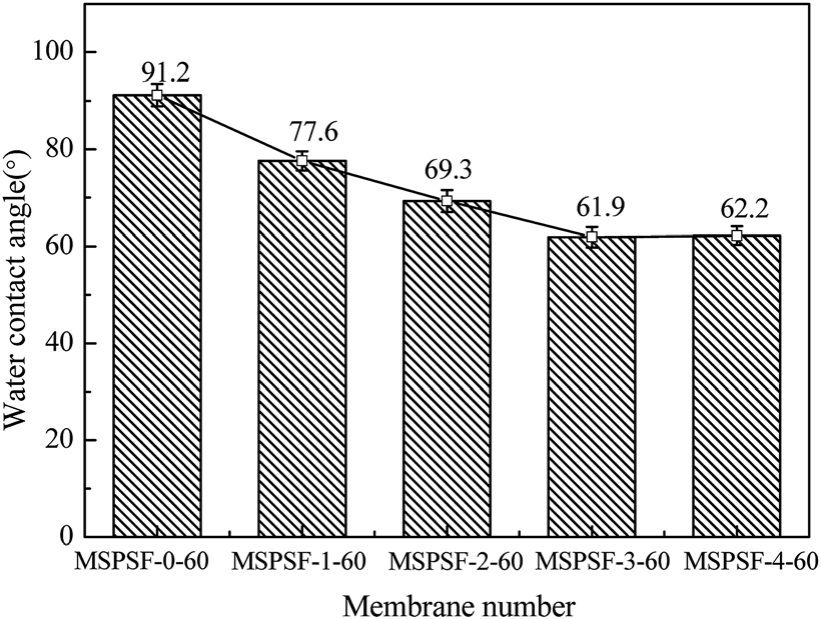
The water contact angle of the PES/SPSF flat-sheet membrane.

The effect of SPSF content on static pure water contact angle was displayed in Fig. 11, the static pure water contact angle was measured by testing the top surface of the PES/SPSF flat-sheet membrane. The static pure water contact angle of MSPSF-0-60, MSPSF-1-60, MSPSF-2-60, MSPSF-3-60 and MSPSF-4-60 were 91.2°, 77.6°, 69.3°, 61.9° and 62.2°, respectively. The result was mainly because SPSF had sulfonic acid hydroxyl group and SPSF transferred to the top surface of the flat-sheet membrane during membrane formation process. It is well-known that the smaller the contact angle, the better the hydrophilicity of the flat-sheet membrane,^29^ therefore, the SPSF improved the hydrophilicity of the PES/SPSF flat-sheet membrane.

### 3.8 Permeation performance

**Fig. 12:**
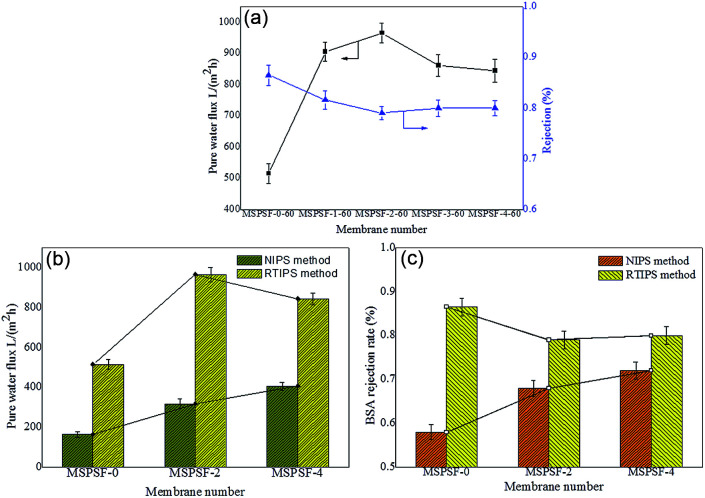
The permeability properties of flat-sheet membrane. (a) The pure water flux and BSA rejection rate of the membrane with SPSF; (b) the pure water flux comparison of NIPS and RTIPS; (c) the BSA rejection rate comparison of NIPS and RTIPS.

The influence of SPSF and membrane formation mechanism on the permeation properties and BSA rejection rate were illustrated in Fig. 12. The pure water flux (*J*_w_) of the flat-sheet membrane, as illustrated in Fig. 12a, showed a first increasing and then decreasing trend with the increase of SPSF, this result matched with the mean pore size (*r*_m_) listed in Table 4. The pure water flux of PES/SPSF membrane was much higher than that of pure PES membrane, however, the BSA retention decreased slightly, this was attributed to homogeneous porous surface and spongy like cross-section obtained by RTIPS method. The maximum pure water flux (966 L m^−2^ h^−1^) and BSA rejection rate (79%) were obtained from the PES/SPSF flat-sheet membrane with 2 wt% SPSF.

**Table 4:** The comparison with other membranes

Membranes	Preparation method	Water bath temperature (K)	Flux (L m^−2^ h^−1^)	*R*_e_ (%)	Reference
PES/DMAc/DEG	VIPS	313	1090 ± 35	10.1 ± 0.3 (BSA, 67 000)	30
PES/DMAc/PEG200	RTIPS	298	1040 ± 56	38.6 ± 3.1 (BSA, 67 000)	10
PSF/HBPE/DMAc/PEG400	RTIPS	298	375 ± 17	≥90 ± 3 (DEX, 1440 kDa)	11
PSF–PANI/TiO_2_/NMP	NIPS	298	187 ± 11	—	31
PES/PES-*b*-PSBMA/DMAc	NIPS	298	119 ± 11	80 ± 1.1 (BSA, 67 000)	32
PES/SPSF/O-MWCNT	NIPS	313	553 ± 21	100 ± 1.8 (BSA, 67 000)	33
PES/SPSF/DMAc/DEG	RTIPS	323	966 ± 45	79.2 ± 2 (BSA, 67 000)	This study

When the coagulation water bath temperature was 25 °C, the membrane formation mechanism underwent NIPS process, while the membrane formation mechanism followed RTIPS process when the coagulation water bath temperature was 60 °C. The pure water flux and BSA rejection rate of the flat-sheet membrane, as shown in Fig. 12b and c, were both higher than that of the membrane by NIPS method.

Based on these results, it could be seen clearly that PES/SPSF flat-sheet membrane by RTIPS process could obtain high pure water flux as well as BSA interception kept at a high value. RTIPS method could be applied as a new method for high pure water flux membrane preparation.

### 3.9 Antifouling properties

**Fig. 13:**
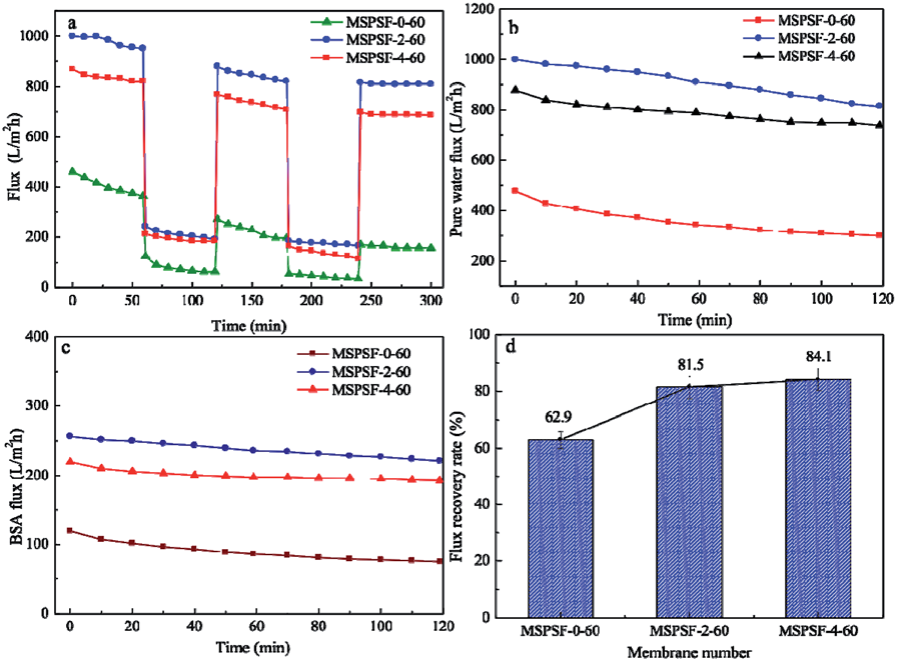
Permeability of membrane in long-term operation. (a) Flux variation of membranes between the pure water flux and BSA solution flux; (b) and (c) the pure water flux and BSA rejection rate with time; (d) flux recovery ratios of membranes.

Fig. 13 showed time-dependent flux during fouling measurement of PES/SPSF flat-sheet membrane using 0.3 g L^−1^ BSA solution as protein foulant at 0.1 MPa. The pollution resistance cycle experiment included three pure water filtration and two fouling stages with 0.3 g L^−1^ BSA solution. The contaminated membrane was backwashing with pH = 10 alkali solution after each fouling stage. It could be seen in Fig. 13a that the flux attenuation rate of PES/SPSF membrane was lower than that of pure PES membrane, this flux tendency was an indicative of membrane fouling degree. A more quantifiable approach was employed to measure membrane antifouling property by the flux recovery ratio (FRR), higher FRR value indicated better anti-pollution performance. The results given in Fig. 13d clearly illustrated that the FRR values of MSPSF-2-60 and MSPSF-4-60 were higher (>80%) than that of MSPSF-0-60 (62.9%), moreover, the FRR increased with the increased SPSF content. The improving antifouling properties of PES/SPSF membranes were attributed to the improvement hydrophilicity by SPSF, these hydrophilic groups prevented the attachment of foulants. The BSA flux and pure water flux with time in Fig. 13b and c also indicated better hydrophilicity with the addition of SPSF. Therefore, the results indicated that the inclusion of SPSF in PES flat-sheet membranes leaded to improved antifouling properties towards protein contaminants.

### 3.10 Comparison with other membranes

In comparison, the PES/SPSF membrane prepared in this study was compared with other membranes in available literature in Table 4. High pure water flux and BSA rejection rate could be acquired simultaneously by RTIPS process. Moreover, the anti-fouling property was enhanced by SPSF. In addition, after two stages of anti-pollution cycle, the initial pure water flux of up to 84.1% resumed. In general, PES/SPSF flat-sheet membrane by RTIPS method was better for application of water treatment.

## 4 Conclusion

This work investigated the effect of SPSF and membrane formation mechanism (NIPS or RTIPS method) on the properties of PES/SPSF flat-sheet membrane. The hydrophilicity of the prepared membrane was enhanced by the presence of SPSF. Membranes containing SPSF exhibited superior antifouling property compared to pristine PES/SPSF membrane. The hydrogen bonding interaction between SPSF, PES and DEG led to an increase in the viscosity of the cast solution. Cloud point increased with increasing SPSF content, moreover, the water contact angle decreased from 91.2° to 62.2° with addition of SPSF. The pure water flux (*J*_w_) and BSA rejection rate (*R*_BSA_) of the flat-sheet membrane first increased and then decreased with the addition of SPSF, the maximum pure water flux (966 L m^−2^ h^−1^) and BSA rejection rate (79.2%) were obtained by the PES/SPSF membrane with 2 wt% SPSF.

As for the influence of membrane formation mechanism (NIPS or RTIPS method) on the performance of the flat-sheet membrane. The membrane preparation mechanism turned NIPS into RTIPS process, as well as dense skin top surface changed into homogeneous porous top surface when the membrane formation mechanism was NIPS method. With regard to the membrane prepared by RTIPS method, the pure water flux and BSA rejection rate were both higher than that by NIPS process. This confirmed that the property of the flat-sheet membrane prepared by RTIPS was better than that by NIPS.

Generally, PES/SPSF flat-sheet membrane prepared by RTIPS method displayed higher pure water flux, BSA rejection rate and anti-pollution properties. This study offered a new attempt to prepare flat-sheet membrane with good performance. The PES/SPSF membrane displayed promising features for the fabrication of fabric-free support substrate with enhanced performance as well as for ultrafiltration membranes use in wastewater treatment.

## Conflicts of interest

The authors declare no competing financial interest.

## Supplementary Material
